# Incidence of postoperative hypothermia and its risk factors in adults undergoing orthopedic surgery under brachial plexus block: A retrospective cohort study

**DOI:** 10.7150/ijms.55023

**Published:** 2021-03-21

**Authors:** Choon-Kyu Cho, Minhye Chang, Tae-Yun Sung, Young Seok Jee

**Affiliations:** 1 Department of Anaesthesiology and Pain medicine, Konyang University Hospital, Konyang University College of Medicine, Daejeon, Korea.; 2 Department of Anaesthesiology and Pain medicine, Konyang University Hospital, Myunggok Medical Research Center, Konyang University College of Medicine, Daejeon, Korea.

**Keywords:** hypothermia, incidence, risk factors, brachial plexus block

## Abstract

Postoperative hypothermia increases patient mortality and morbidity. However, the incidence of, and risk factors for, postoperative hypothermia in patients undergoing surgery under brachial plexus block (BPB) as the primary method of anesthesia remain unclear. This study aimed to determine the incidence of, and risk factors for, postoperative hypothermia in patients undergoing surgery under BPB. We retrospectively analyzed 660 patients aged ≥ 19 years who underwent orthopedic surgery under BPB in our hospital between October 2014 and October 2019. Postoperative hypothermia was defined as a tympanic membrane temperature < 36 °C when the patient arrived in the post-anesthesia care unit. Multivariate logistic regression analysis was performed to identify the independent risk factors for postoperative hypothermia. Postoperative hypothermia was observed in 40.6% (268/660) of patients. Independent risk factors for postoperative hypothermia were lower baseline core temperature before anesthesia (odds ratio [OR] 0.355; 95% confidence interval [CI] 0.185-0.682), alcohol abuse (OR 2.658; 95% CI 1.105-6.398), arthroscopic shoulder surgery (OR 2.007; 95% CI 1.428-2.820), use of fentanyl (OR 1.486; 95% CI 1.059-2.087), combined use of midazolam and dexmedetomidine (OR 1.816; 95% CI 1.268-2.599), a larger volume of intravenous fluid (OR 1.001; 95% CI 1.000-1.002), and longer duration of surgery (OR 1.010; 95% CI 1.004-1.017). Postoperative hypothermia is common in adult patients undergoing orthopedic surgery under BPB. The risk factors identified in this study should be considered to avoid postoperative hypothermia in these patients.

## Introduction

The incidence of postoperative hypothermia has been reported to be 8-90% depending on the type of surgery, method of anesthesia, patient's age, definition of hypothermia, measurement site and thermometer type used 1-3. Hypothermia can increase the risk for myocardial ischemia/infarction, ventricular arrhythmias, impaired mentation, coagulopathy, increased blood loss, need for allogenic transfusion, surgical site infection, venous stasis, pressure ulcer, prolonged drug effect, prolonged hospitalization, and mortality 4-6.

To prevent adverse events due to hypothermia, it is important to monitor body temperature carefully and identify risk factors for hypothermia. However, core-temperature monitoring of patients undergoing regional anesthesia is seldom performed due to patients' discomfort as a result of the invasiveness of the insertion of a measurement probe and the absence of a convenient and reliable measurement site 7. In studies conducted in patients undergoing general anesthesia or neuraxial anesthesia, female sex, older age, low body mass index, low preoperative body temperature, low operating-room temperature, major operation, longer duration of surgery, amount of intravenous fluid, high level of neuraxial anesthesia and anesthetic technique were associated with the occurrence of hypothermia 2,3,5,8,9. However, risk factors for hypothermia in patients undergoing surgery under brachial plexus block (BPB) as the main method of anesthesia remain unclear. The risk factors for hypothermia may be different in patients receiving BPB because the effect of BPB on central thermoregulatory control and the extent of nerve block on vasomotor tone differ from those of general anesthesia or neuraxial anesthesia. Identification of the incidence of, and risk factors for, postoperative hypothermia in these patients can provide evidence regarding the necessity of temperature monitoring in patients undergoing surgery under peripheral nerve block, such as BPB, and help to prevent hypothermia. Therefore, we designed this study to determine the incidence of and risk factors for postoperative hypothermia in patients receiving BPB as the primary method of anesthesia.

## Methods

This retrospective study was approved by the institutional review board of Konyang University Hospital, Daejeon, Korea (approval number KYUH 2019-11-007-002 on 20 November 2019; Chairperson Prof. JW Son), and was registered with the Korea Clinical Research Information Service (http://cris.nih.go.kr; approval number, KCT 0005332). Informed consent was waived due to the retrospective nature of the study. We retrospectively reviewed the medical records of patients aged ≥ 19 years who underwent orthopedic surgery under BPB as the primary method of anesthesia in our hospital between October 2014 and October 2019. Cases of conversion to general anesthesia after BPB and omission of covariate data records, as well as failure to record body temperature, were excluded from this study.

All patients arrived in the pre-operative holding area without premedication in the hospital ward. In our institution, the ambient temperature in the pre-operative holding area and post-anesthesia care unit (PACU) is maintained at 22-25 °C, while the ambient operating-room temperature is maintained at 21-24 °C. When being moved from the preoperative holding area to the operating room and from the operating room to the PACU, the patients lay in bed and were covered with cotton blankets. The transfer time was within 1 min. The ambient temperature in the corridors to and from the operating room was controlled to be the same as in the operating room.

After arrival in the operating room, routine monitoring was started, including electrocardiogram, noninvasive blood pressure (NIBP), and pulse oximetry. BPB was performed under ultrasound and nerve stimulator guidance by an anesthesiologist using 20-25 ml of 0.5% ropivacaine prepared by mixing 0.75% ropivacaine (20 ml) and normal saline (10 ml). The BPB approach was determined at the discretion of the anesthesiologist, considering the surgical site and the patient's condition. The adequacy of the block was confirmed using a pin-prick test. After BPB, oxygen (6l/min) was supplied *via* a facemask, and respiration was monitored by end-tidal carbon dioxide in all patients. Blood pressure was controlled using a hypotensive agent (nicardipine) or vasopressors (phenylephrine, ephedrine) to maintain a target pressure within 80-120% of the systolic blood pressure measured before BPB or mean blood pressure of 60-110 mmHg. Persistent bradycardia was treated with anticholinergics (atropine, glycopyrrolate). Sedatives (midazolam, dexmedetomidine, propofol) and/or analgesics (morphine, fentanyl, pethidine) were chosen and administered intravenously at the patient's request or at the discretion of the attending anesthesiologist. The level of sedation was intended to be moderate sedation 10.

No patient received prewarming using a forced-air warming device. In addition, no active warming using a forced-air warming device was applied during the BPB procedure. However, during surgery, active warming was applied using a forced-air warming blanket (Bair Hugger^TM^ Lower-body Cover Model 52500, Heater Model 505; Arizant Healthcare Inc., USA) placed over the patient from the level of the xiphoid process to the feet and covered with a surgical drape, unless refused by the patient; tympanic membrane temperature measured in the pre-operative holding area was > 37 °C. The forced-air warming device was set at 38 °C throughout the surgery. All intravenous fluids and irrigation solutions were administered at room temperature without warming.

Following our institutional protocol and regardless of the method of anesthesia, all patients' temperatures were measured by an anesthesiology nurse on arrival in the pre-operative holding area and at PACU admission using an infrared tympanic thermometer (Thermoscan IRT 4020, Braun GmbH, Kronberg, Germany; accurate to ± 0.2 °C for patient temperatures in the range 35.5-42 °C ± 0.3 °C for patient temperatures < 35.5 °C). However, monitoring of intraoperative core-temperature in patients undergoing surgery under BPB is not a standard of care in our institution; in these patients, intraoperative temperature monitoring is conducted only in some patients, without obvious criteria. The tympanic membrane temperature measured in the pre-operative holding area was considered the baseline core temperature before anesthesia, and postoperative hypothermia was defined as a tympanic membrane temperature of less than 36 °C at PACU admission. In addition, the severity of hypothermia was classified as mild (35-35.9 °C), moderate (34-34.9 °C), or severe (≤ 34 °C) 3.

The following data were collected and analyzed: baseline core temperature before anesthesia, age, sex, weight, height, body mass index, American Society of Anesthesiologists' physical status, comorbidities, smoking history, alcohol abuse (individuals imbibing on average 3-4 drinks a day at least four or more times a week), the approach of BPB, BPB site (left or right side), type of surgery (non-arthroscopic surgery and arthroscopic shoulder surgery), use of intraoperative forced air-warming device, administered vasopressors, hypotensive agent, anticholinergics, analgesics, sedatives, amount of intravenous fluid administered during surgery, blood loss estimated by the surgeon, and duration of surgery.

### Statistical analysis

Statistical analysis was performed using SPSS Statistics™ software (ver. 18.0 for Windows; IBM SPSS Inc., Chicago, IL, USA). For data analysis, patients with postoperative hypothermia (temperature < 36 °C at PACU admission) were included in the hypothermia group, while those with normothermia (temperature 36-38°C at PACU admission) were included in the normothermia group 11.

The comparison of pre- and postoperative tympanic membrane temperatures was analyzed using the Wilcoxon signed rank test. In the univariate analysis, continuous variables were analyzed using Student's *t*-test or the Mann-Whitney *U* test as appropriate, after assessing the data distribution using the Kolmogorov-Smirnov test. Categorical variables were analyzed using the χ^2^ test or Fisher's exact test, as appropriate. All variables with *P*-values < 0.1 in the univariate analysis were included in the multivariate analysis using backward binary stepwise logistic regression to determine independent risk factors of perioperative hypothermia. The appropriateness of the model was assessed using the Hosmer-Lemeshow test. A *P*-value < 0.05 was considered to indicate statistical significance.

## Results

**Figure 1:**
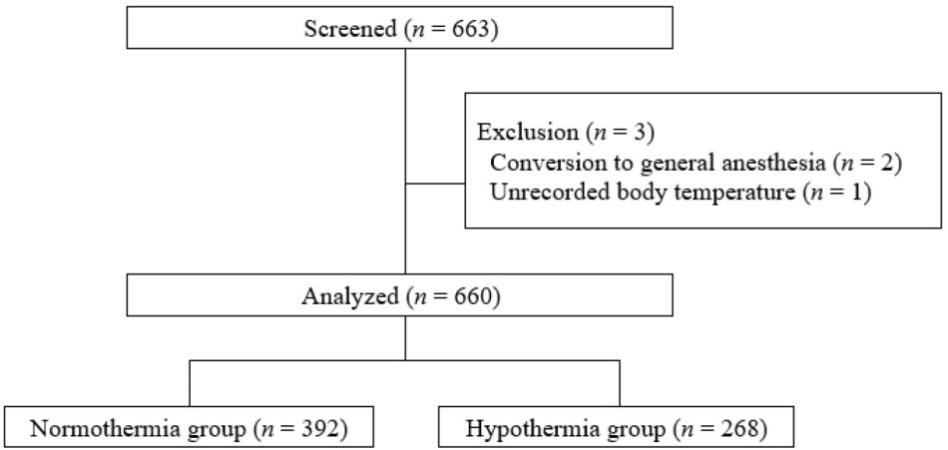
Flow chart of the study.

In total, 663 patients were enrolled following the inclusion criteria of this study. Of them, three patients were excluded: two because of conversion to general anesthesia after BPB and one because body temperature was not recorded at PACU admission. Thus, 660 patients aged from 20 to 93 years (median, 65 years; interquartile range: 57-75 years) were included in this analysis (Fig. 1). Among the included patients, 268 with a tympanic membrane temperature < 36°C at PACU admission were included in the hypothermia group, and the remaining 392 were included in the normothermia group. The incidence of postoperative hypothermia was 40.6% (268/660). Among those cases, the severity of hypothermia was mild in 98.5% (264/268) and moderate in 1.5% (4/268); severe hypothermia did not occur. The median [interquartile range] tympanic membrane temperature upon arrival in the PACU was 36.2°C [36.0-36.4°C] in the normothermia group and 35.7°C [35.6-35.9°C] in the hypothermia group (*P* < 0.001). Postoperative tympanic membrane temperature was significantly lower compared with baseline core temperature before anesthesia in the both groups (both *P* < 0.001).

Tables 1 and 2 show patient- and procedure-related risk factors for postoperative hypothermia after BPB, respectively. In univariate analysis, sex, alcohol abuse, type of surgery, use of fentanyl, combined use of midazolam and dexmedetomidine, amount of intraoperative fluid, and duration of surgery differed significantly between the two groups (*P* < 0.05). In addition to these seven variables, two variables (baseline core temperature before anesthesia and use of phenylephrine) associated with postoperative hypothermia (*P* < 0.1) in the univariate analysis were included in the multivariate logistic regression analysis. Multivariate analysis showed that lower baseline core temperature before anesthesia (odds ratio [OR] 0.355; 95% confidence interval [CI] 0.185-0.682), alcohol abuse (OR 2.658; 95% CI 1.105-6.398), arthroscopic shoulder surgery (OR 2.007; 95% CI 1.428-2.820), use of fentanyl (OR 1.486; 95% CI 1.059-2.087), combined use of midazolam and dexmedetomidine (OR 1.816; 95% CI 1.268-2.599), larger amounts of intravenous fluid (OR 1.001; 95% CI 1.000-1.002), and longer duration of surgery (OR 1.010; 95% CI 1.004-1.017) were independent risk factors for postoperative hypothermia (Table 3). The Hosmer-Lemeshow test confirmed the validity of the model (*P* = 0.800).

## Discussion

This study demonstrated that postoperative hypothermia is common in patients undergoing surgery under BPB. In this study, 40.6% of patients who underwent surgery under BPB had a tympanic membrane temperature of less than 36°C at PACU admission. This implies that temperature monitoring should be performed as a standard of care to prevent adverse events due to hypothermia, even in patients undergoing surgery under BPB. Independent risk factors for postoperative hypothermia included lower baseline core temperature before anesthesia, alcohol abuse, arthroscopic shoulder surgery, use of fentanyl, combined use of midazolam and dexmedetomidine, larger amounts of intravenous fluid, and longer duration of surgery.

Both general and regional anesthesia impair central thermoregulation, although the extent of impairment is less with regional anesthesia 6. Internal core (e.g., thorax, abdomen) to peripheral (e.g., arms, legs) redistribution of body heat is the most important cause of hypothermia after general or regional anesthesia 5,12. In the case of regional anesthesia, redistribution of body heat is caused not only by centrally mediated vasodilation but also, and more importantly, by direct inhibition of vasoconstriction (and shivering) in the regionally blocked area 6,12. However, whereas neuraxial anesthesia such as spinal or epidural anesthesia results in nerve block in both lower extremities, which constitute most of the peripheral thermal compartment, BPB causes nerve block only in one upper limb; thus, the magnitude of heat loss due to redistribution may be less in BPB than in neuraxial anesthesia.

In this study, postoperative hypothermia was common (40.6%), although BPB was performed as the primary anesthesia method and active intraoperative warming was applied in most (83.5%) patients. Forced-air warming is effective for maintaining normothermia by preventing intraoperative heat loss from the skin surface through radiation and convection 11,13. The reason why the severity of hypothermia was relatively mild in this study is that a significant number of patients underwent intraoperative forced-air warming. However, in several studies 14-16, active intraoperative warming alone was often ineffective for preventing perioperative hypothermia. Hypothermia was observed in more than 50% of our patients who received general or neuraxial anesthesia despite the use of intraoperative forced-air warming devices. This finding indicates that additional strategies are needed to prevent postoperative hypothermia.

In addition to the method of anesthesia, patient- and procedure-related factors can also affect development of hypothermia 1-3,8,9. Similar to previous studies 8,17, lower baseline core temperature before anesthesia, arthroscopic shoulder surgery, larger amounts of intravenous fluid, and longer duration of surgery were associated with an increased risk for hypothermia in this study. The baseline core temperature before anesthesia implies the heat content of peripheral tissue and the core-to-peripheral tissue temperature gradient, which determines the extent of redistribution 17. During arthroscopic shoulder surgery, large amounts of irrigation solution are used to ensure the surgical field of view. Although the effects of the use of warmed irrigation solution on the prevention of hypothermia are inconsistent 18,19, un-warmed irrigation solution is absorbed into the systemic circulation and decreases body temperature 20. Intravenous administration of more than 1 liter of unwarmed fluid increases the risk of hypothermia by nearly three times compared to administration of less than 1 liter of unwarmed fluid 8. The slight increase in the risk (odds ratio = 1.001) of hypothermia in this study may have been due to the small amounts (median = 300 ml) of fluid administered to both groups. The increased risk for hypothermia with a long operation time may have been due to increased blood loss and fluid demand, as well as increased heat loss by radiation and convection from the skin and by evaporation from within surgical incisions 2,13.

Analgesics and sedatives are frequently supplemented during BPB to improve acceptance of the block and reduce patient discomfort. Most of these drugs (e.g., alfentanil, propofol, dexmedetomidine), except midazolam, produce a marked dose-dependent reduction in the thresholds of vasoconstriction and shivering 12; however, midazolam at clinical doses slightly impairs thermoregulatory control 21. Although fentanyl was a protective factor against hypothermia in a previous study 1, this was probably due to limited sample size (n = 78). Alfentanil linearly reduces the thresholds of vasoconstriction and shivering, and it is assumed that other pure μ-opioid receptor agonists, such as fentanyl, have the same effect 22. In our study, the use of dexmedetomidine or midazolam alone did not increase the risk for hypothermia in patients receiving BPB, but combined use of the two increased the risk for hypothermia almost two-fold. Future studies are needed to determine whether the effect of combined use of midazolam and dexmedetomidine on thermoregulatory impairment is additive or synergistic.

In this study, alcohol abuse was the most important risk factor associated with postoperative hypothermia. To our knowledge, no study has found alcohol abuse to be a risk factor for hypothermia. Possible explanations are as follows. Acutely, alcohol contributes to the development of hypothermia by inducing peripheral vasodilation and inhibiting plasma vasopressin release, whereas chronic alcohol abuse may be associated with sympathetic dysfunction and hypothalamic lesions (e.g., Wernicke's encephalopathy), which can lead to impairment of thermoregulation 23. In addition, chronic alcoholics tend to have a lower body weight compared to healthy social drinkers due to fat mass reduction 24. Fat tissue has an insulating function, and lower body weight is a risk factor for hypothermia 13,25. Perioperative thiamine supplementation may help to prevent hypothermia in patients who abuse alcohol 23.

This study has several limitations. First, in several studies 2,9,13, older age (≥ 60 years) was a risk factor for inadvertent hypothermia. The median age [interquartile range] of the subjects in this study was 65 [57-75] years, and a large proportion of patients were aged > 60 years. This may have contributed to an increase in the overall incidence of postoperative hypothermia in this study. Second, the ambient operating-room temperature could not be analyzed. The effect of ambient operating-room temperature on patients' core temperatures has been inconsistent among studies 8,9,26. However, in a recent study of patients undergoing general anesthesia, the operating-room temperature had a negligible effect on patients who were warmed with forced-air and a small effect on patients who were passively insulated 26. In our study, 83.5% (551/660) of the patients received active warming with a forced-air blanket during surgery; thus, the effect of operating-room temperature on the patients' core temperatures would have been insignificant. Third, core temperatures were evaluated using an infrared tympanic thermometer. Although infrared tympanic thermometers are widely used to measure core temperature in clinical practice, especially in patients undergoing regional anesthesia 27, the accuracy of such thermometers has been questioned 13. Because infrared tympanic thermometers often measure only the temperature of the aural canal or the area near the temporal artery, and not that of the tympanic membrane, temperatures measured using these thermometers may show differences > 1°C from the actual core temperature 12,28. Accuracy can also be affected by the user, structure of the aural canal, skin temperature on the face, and exudate and fluid in the ears 12,29. In recent studies 30,31, new non-invasive thermometers, such as the 3M SpotOn® and Dräger Tcore®, have shown high accuracy and precision for measuring core temperature in patients undergoing general anesthesia. These thermometers are expected to be useful for measuring core temperature in patients under regional anesthesia. Finally, active warming of the skin surface before induction of anesthesia (i.e., prewarming) is the most effective method for preventing hypothermia in patients undergoing general anesthesia or neuraxial anesthesia 11. However, in this study, it was not possible to analyze whether prewarming acts as a protective factor against hypothermia because no patient received prewarming. As the efficacy of active prewarming of patients receiving BPB has not been studied, future studies are needed to determine its efficacy in the prevention of hypothermia in patients receiving BPB.

In conclusion, the incidence of postoperative hypothermia in patients receiving BPB was high. Lower baseline core temperature before anesthesia, alcohol abuse, arthroscopic shoulder surgery, use of fentanyl, combined use of midazolam and dexmedetomidine, larger amounts of unwarmed fluid, and longer surgery were associated with the development of postoperative hypothermia. To prevent complications due to postoperative hypothermia in patients undergoing surgery under BPB, careful intraoperative monitoring of body temperature and additional interventions to avoid the identified risk factors should be considered.

## Figures and Tables

**Table 1:** Univariate analysis: patient-related risk factors for postoperative hypothermia after brachial plexus block

Variable	Normothermia (*n* = 392)	Hypothermia (*n* = 268)	*P*
Baseline core temperature, °C	36.8 [36.6-36.9]	36.7 [36.6-36.9]	0.053
Age, years	66 [56-75]	64 [57-74]	0.663
Sex, male/female	154/238	127/141	0.039
Weight, kg	60 [52.5-69]	61.2 [54-70]	0.661
Height, cm	157 [150-165]	158 [151-167]	0.429
Body mass index (kg/m^2^)	24.4 [22.2-26.7]	24.1 [22.1-26.5]	0.511
ASA physical status (I/II/III)	33/262/97	23/192/53	0.322
**Patient comorbidities**			
Hypertension	184 (46.9%)	129 (48.1%)	0.763
Diabetes mellitus	96 (24.5%)	59 (22.0%)	0.461
Hypothyroidism	3 (0.8%)	4 (1.5%)	0.450
Pulmonary disease	14 (3.6%)	13 (4.9%)	0.415
Current smokers	50 (12.8%)	39 (14.6%)	0.507
Alcohol abuse	9 (2.3%)	17 (6.3%)	0.013

Values are presented as median [interquartile ranges], numbers, or number (%). ASA, American Society of Anesthesiologists.

**Table 2:** Univariate analysis: procedure-related risk factors for postoperative hypothermia after brachial plexus block

Variable	Normothermia (*n* = 392)	Hypothermia (*n* = 268)	*P*
**Approach of block**			0.510
Interscalene	308 (78.6%)	221 (82.5%)	
Supraclavicular	18 (4.6%)	12 (4.5%)	
Infraclavicular	9 (2.3%)	3 (1.1%)	
Axillary	57 (14.5%)	32 (11.9%)	
BPB on left/right side	162/230	112/156	0.905
**Type of surgery**			< 0.001
Non-arthroscopic surgery	193 (49.2%)	91 (34.0%)	
Arthroscopic shoulder surgery	199 (50.8%)	177 (66.0%)	
Forced-air warmer	322 (82.1 %)	229 (85.4%)	0.261
**Vasopressors**			
Phenylephrine	9 (2.3%)	13 (4.9%)	0.073
Ephedrine	37 (9.4%)	36 (13.4%)	0.108
Hypotensive agent (nicardipine)	34 (8.7%)	18 (6.7%)	0.359
**Anticholinergics**			
Atropine	2 (0.5%)	3 (1.1%)	0.401
Glycopyrrolate	0 (0%)	1 (0.4%)	0.406
**Analgesics**			
Fentanyl	211 (53.8%)	167 (62.3%)	0.030
Morphine	4 (1.0%)	4 (1.5%)	0.721
Pethidine	0 (0%)	2 (0.7%)	0.165
Fentanyl + morphine	1 (0.3%)	1 (0.4%)	> 0.999
**Sedatives**			
Midazolam	115 (29.3%)	73 (27.2%)	0.558
Dexmedetomidine	80 (20.5%)	47 (17.5%)	0.350
Propofol	27 (6.9%)	17 (6.3%)	0.783
Midazolam + dexmedetomidine	94 (24.0%)	101 (37.7%)	< 0.001
Fluids, mL	300 [200-400]	300 [200-450]	0.001
Transfusion	0 (0%)	0 (0%)	NA
Estimated blood loss, mL	10 [5-15]	10 [5-15]	0.807
Duration of surgery, min	60 [45-85]	75 [55-95]	< 0.001

Values are presented as numbers, number (%), or median [interquartile ranges]. BPB, brachial plexus block; NA, not applicable.

**Table 3:** Multivariate logistic regression analysis: independent risk factors of postoperative hypothermia after brachial plexus block

Variable	Odds ratio	95% CI	*P*
Baseline core temperature	0.355	0.185-0.682	0.002
Alcohol abuse	2.658	1.105-6.398	0.029
Arthroscopic shoulder surgery	2.007	1.428-2.820	< 0.001
Fentanyl	1.486	1.059-2.087	0.022
Midazolam + dexmedetomidine	1.816	1.268-2.599	0.001
Fluid administered	1.001	1.000-1.002	0.035
Duration of surgery	1.010	1.004-1.017	0.002

CI, confidence interval.
